# Impact of injection scheduling on CO_2_ migration and trapping efficiency in the Johansen formation

**DOI:** 10.1371/journal.pone.0340048

**Published:** 2025-12-31

**Authors:** Hai T. Nguyen

**Affiliations:** 1 Laboratory of Applied Physics, Science and Technology Advanced Institute, Van Lang University, Ho Chi Minh City, Vietnam; 2 Faculty of Applied Technology, Van Lang School of Technology, Van Lang University, Ho Chi Minh City, Vietnam; Sichuan University of Science and Engineering, CHINA

## Abstract

Injection scheduling is increasingly considered as an operational lever for optimizing carbon dioxide (CO_2_) storage in saline aquifers, yet its long-term impact on trapping efficiency at field scale remains uncertain. This study employs a field-calibrated vertical equilibrium model of the Johansen formation, offshore Norway, to compare four injection strategies: constant, ramped, pulsed, and low steady, under equal total injected mass. Simulations span 1000 years, including both injection and post-injection phases, to evaluate plume migration, bottom hole pressure (BHP) evolution, and partitioning among residual and solubility trapping. Results show that while injection schedule significantly influences short-term injectivity and peak BHP with differences up to 80 bar, its effect on millennial-scale trapping efficiency is negligible. By 1000 years, all scenarios converge to a similar distribution, with 57–58% dissolved in brine, 30–34% immobilized by residual trapping, and 8–9% persisting as a mobile plume, with inter-schedule differences less than 2% of the total injected mass. These findings indicate that, in a laterally open and well-connected aquifer such as Johansen, long-term storage security is governed primarily by reservoir properties and dissolution dynamics rather than by operational schedule. Consequently, injection scheduling should be regarded as a tool for short-term pressure management and infrastructure safety, not as a determinant of ultimate storage performance. This distinction provides practical guidance for designing CO_2_ storage projects and regulatory assessments of long-term containment.

## Introduction

Anthropogenic carbon dioxide (CO_2_) emissions remain a primary driver of global climate change, posing major challenges to environmental stability and sustainable development [1–3]. Geological carbon storage is recognized as an essential mitigation option within integrated carbon management strategies [4–6]. Among available storage options, deep saline aquifers offer extensive capacity and favorable long-term containment conditions [7–9]. Despite increasing attention to their large-scale deployment, the influence of operational design, particularly injection scheduling, on long-term storage performance remains insufficiently understood at the field scale.

Accurate modeling of CO_2_ plume migration and containment is essential for the safe, large-scale CCS deployment. However, these simulations must span spatial scales of tens of kilometers and temporal scales of centuries or more. While fully three-dimensional (3D) multiphase flow models offer detailed local predictions, their computational cost limits their practicality for long-term or basin-scale analysis [10–13]. Even simplified 3D models may underestimate plume migration velocities when numerical resolution is misaligned with formation scale. These challenges are further compounded by limited high-resolution geological data in many deep saline formations [14].

To address these issues, vertical equilibrium (VE) models have gained traction as computationally efficient alternatives. By assuming rapid buoyant segregation of CO_2_ and brine, VE models reduce the governing equations to a depth-integrated two-dimensional (2D) approximation that captures dominant large-scale migration behavior [12,15]. Sharp-interface VE models have successfully reproduced long-term CO_2_ dynamics in systems such as the Utsira and Johansen formations with substantially reduced computational demands. However, these models also introduce physical simplifications that warrant scrutiny.

A principal limitation of sharp-interface VE models is their inability to resolve the capillary transition zone, where CO_2_ and brine coexist in the pore space and capillary forces control fluid distribution and trapping [16–19]. In reality, fluid properties vary with pressure, temperature, and saturation, producing nonlinear hysteresis in capillary pressure and relative permeability that can significantly affect long-term trapping and dissolution [20–22]. Caprock rugosity, small-scale geological heterogeneity, and pressure-induced geomechanical responses further complicate predictions of injectivity, containment, and trapping security [22–25].

Beyond geological and physical controls, injection strategy is a key operational parameter that influences plume shape, pressure evolution, and trapping performance. Variable-rate and cyclic injection schemes have shown promise in enhancing residual and solubility trapping under favorable conditions. For example, certain variable-rate profiles (including cyclic) can increase residual trapping in cores, translation to field scale remains uncertain. [26,27]. Yet translating laboratory or synthetic findings to field-scale behavior remains challenging due to scale effects, heterogeneity, and incomplete parameterization [28]. Moreover, while cyclic schemes are often proposed for pressure management, their effectiveness for trapping varies by context, and water-alternating-gas approaches may offer more reliable outcomes in some settings [29]. Wettability and pore geometry also modulate these effects by altering phase connectivity and flow regimes.

This study addresses the unresolved question of how injection scheduling influences CO_2_ migration and trapping over millennial timescales under field-calibrated conditions. By applying equal-mass scheduling strategies within a validated Johansen VE model, the analysis clarifies the operational versus geological controls on long-term storage security, representing a necessary step toward risk-informed CCS deployment.

## Methodology

This study uses a field-calibrated VE model of the Johansen formation to simulate long-term CO_2_ injection and migration under four distinct operational schedules. The simulations are implemented within the open-source MRST-CO_2_lab framework, leveraging its efficient depth-integrated solvers to capture basin-scale buoyancy-driven flow over millennial timescales. Results are postprocessed to quantify plume evolution, bottom-hole pressure (BHP) dynamics, and long-term trapping mechanisms.

### Geological model

The Johansen formation is a deep saline aquifer located offshore Norway, beneath the Troll field. The model used here is based on the NPD5 dataset from the MatMoRA project, offering a high-resolution characterization of formation structure and heterogeneity [30–32]. The formation lies between 2200 and 3100 m true vertical depth below sea level, ensuring that injected CO_2_ remains in a supercritical state under in-situ conditions.

The Johansen formation comprises five stacked sandstone layers interbedded with shale, with an average net thickness of approximately 100 m and lateral extents of up to 100 km (N-S) and 60 km (E-W). Porosity averages 25%, and permeability ranges from 64 to 1660 mD. A 2D VE grid of 200 × 150 cells (each approximately 500 × 500 m) represents lateral heterogeneity. Shale interbeds are excluded to simplify vertical structure into a continuous aquifer. The Dunlin Group shale forms the primary caprock, with a sealing fault on the western boundary. Hydrostatic open boundaries are applied to the eastern and southern edges to represent far-field flow.

**Fig 1:**
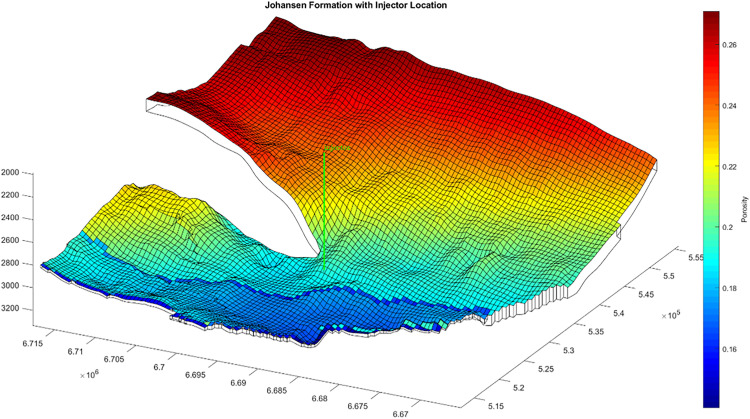
Johansen formation reservoir model used in this study, showing grid resolution and injector placement.

A single vertical injector was placed near the structural center of the model and completed across the main reservoir layers (Fig 1). This location was selected to provide a representative central injection point, enabling symmetric plume development and minimizing boundary effects. The model setup is field-calibrated rather than history-matched. Because no injection data are available for the Johansen formation, model parameters were constrained using geological structure, permeability, and pressure–temperature profiles from the NPD5 and MatMoRA datasets. This calibration reproduces realistic reservoir conditions and provides a representative baseline for evaluating the effects of injection scheduling.

To enable VE modeling, the top surface of the main sandstone unit is extracted and vertically integrated into a 2D domain. This simplification is justified by the high lateral to vertical aspect ratio and strong buoyancy contrast between CO_2_ and brine, which cause vertical segregation to occur much faster than lateral migration. Under these conditions, the VE framework provides an efficient and physically consistent representation of buoyancy-driven migration and long-term trapping. The approach assumes depth-averaged pressure and saturation, instantaneous vertical segregation, and effective representation of vertical heterogeneity through averaged rock properties. These assumptions are appropriate for the geometry and hydraulic behavior of the Johansen formation, which is laterally extensive, gently dipping, and dominated by high permeability sandstone layers.

### VE simulation model

The VE model reduces the full 3D mass and momentum conservation equations to a 2D depth-integrated form, suitable for buoyancy-dominated flow [33,34]. The governing conservation equation for the non-wetting phase (CO_2_) is expressed as:


$$\phi H\frac{\partial S_{g}}{\partial t}+\nabla .u_{g}^{VE}=q_{g}$$ (1)

where φ is porosity, H is local formation thickness, $S_{g}$ is vertically averaged CO_2_ saturation, $u_{g}^{VE}$ is the depth-integrated Darcy velocity of CO_2_, and $q_{g}$ denotes injection source terms. The flux term is given by:


$$u_{g}^{VE}=-\frac{kH}{\mu_{g}}\lambda_{g}^{VE}\left(\nabla p-\rho_{g}g\right)$$ (2)

where k is permeability, $\mu_{g}$ is CO_2_ viscosity, $\lambda_{g}^{VE}$ is the vertically averaged relative permeability of CO_2_, p is pressure, $\rho_{g}$ is CO_2_ density, and g is the gravitational acceleration vector.

Capillary pressure is represented using an inverse capillary-pressure function:


$$P_{c}=P_{e}{\left(\frac{S_{rw}}{S_{g}}\right)}^{\text{2}}$$ (3)

where $P_{e}=\text{5}kPa$ is the capillary entry pressure and $S_{rw}$ and $S_{g}$ denote residual water and gas saturations, respectively. This functional form, implemented through the invPc3D routine in MRST-CO_2_lab, reproduces the characteristic shape of drainage-imbibition curves observed in sand-dominated saline aquifers such as Johansen. The relationship was calibrated to laboratory and field-scale data to reflect realistic entry pressures and saturation ranges under in-situ conditions. Hysteresis is represented using a memory-tracking saturation variable that approximates the residual trapping resulting from drainage-imbibition cycles.

A fully implicit backward Euler scheme is employed for time integration, with 1-year steps during injection (except for the pulsed case, which uses 0.5-year steps) and 10-year steps during post-injection phases. MRST default nonlinear solver tolerances are used (relative residual of 1e-6; 10 max iterations), ensuring robust convergence under strong buoyancy and mobility contrasts.

### Simulation scenarios

**Fig 2:**
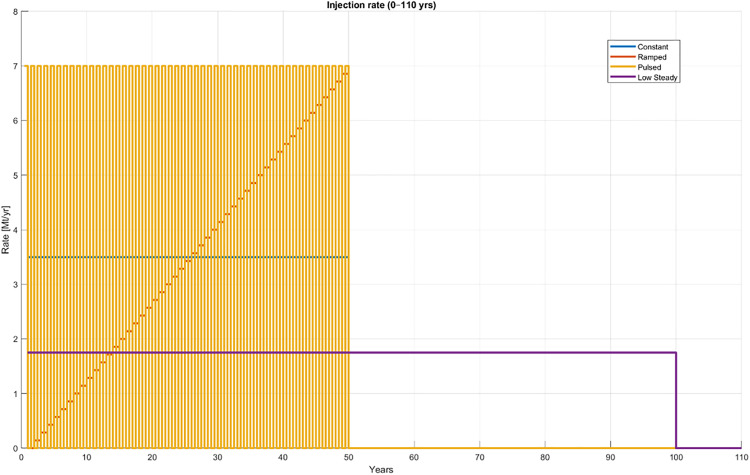
Injection schedules for the four scenarios.

Four injection schedules (Fig 2) were considered to examine the influence of temporal delivery on CO_2_ migration and trapping: (i) Constant rate, (ii) Ramped rate (linear increase), (iii) Pulsed injection (cyclic ON/OFF), and (iv) Low-Steady injection (continuous at half rate).

For the constant, ramped, and pulsed cases, the total injected mass equals Q₀ = 3.5 Mt/year, delivered over a 50-year injection period. For the low-steady case, the same total injected mass Q₀ is delivered over a 100-year injection period at a rate of Q₀/2, so that the cumulative injected mass matches the other schedules (Table 1). The full simulation horizon is 1000 years in all cases. This yields a 950-year post-injection period for the constant, ramped, and pulsed scenarios, and a 900-year post-injection period for the low-steady scenario. Base fluid properties (density, viscosity, solubility) reflect in-situ Johansen conditions. CO_2_ dissolution into brine was included to represent long-term mass transfer between the gas and aqueous phases. Solubility was computed using the equilibrium formulation implemented in MRST-CO_2_lab, which follows the correlation of Spycher and Pruess [35] for CO_2_-H_2_O systems as adopted in the CO2props.m routine [17]. The correlation accounts for the dependence of CO_2_ solubility on pressure, temperature, and salinity, providing equilibrium partitioning between phases. Dissolution was treated as a local equilibrium process, suitable for millennial-scale simulations, with a maximum dissolved mass fraction of about 0.05 at Johansen reservoir conditions (~30MPa, 94^o^C). This design ensures that the total injected CO_2_ mass is identical across schedules, while allowing differences in delivery rate and duration to influence plume growth, pressure evolution, and long-term trapping.

**Table 1:** Injection schedule description.

Scenario	Description
Constant	Constant injection rate at Q_0_ for 50 years
Ramped	Linearly increasing rate from 0 to 2xQ_0_ over 50 years
Pulsed	6 months ON at 2xQ_0_ and 6 months OFF, repeat for 50 years
Low-Steady	Constant rate at Q_0_/2 for 100 years

### Postprocessing and trapping analysis

Following each simulation, the depth-integrated CO_2_ saturation distribution is reconstructed into a pseudo-3D plume geometry by applying formation thickness data. This reconstruction enables spatial visualization of plume extent, height, and migration pathways over time. BHP is continuously monitored at the injection well throughout the simulation to evaluate injectivity performance and assess the pressure mitigation effects of each injection schedule.

To quantify storage security, CO_2_ is classified into four distinct trapping categories. Structural trapping refers to the portion of the plume immobilized beneath the caprock due to topographic confinement. Residual trapping is estimated using the sGmax memory variable, which tracks hysteretic saturation changes during drainage–imbibition cycles. Solubility trapping is calculated based on local thermodynamic equilibrium between CO_2_ and formation brine in contact regions. Any remaining mobile CO_2_ is also accounted for as the untrapped fraction. Although structural trapping is one of the principal storage mechanisms in many CO_2_ storage settings, it does not occur in the Johansen VE model used here. The laterally open aquifer representation with hydrostatic boundaries provides no closed structural highs beneath the caprock, and therefore, no structural immobilization develops. The final distribution across these four categories is evaluated at the end of the 1000-year simulation period, providing a comparative basis for assessing long-term trapping efficiency and storage security among the different injection scenarios.

The vertically integrated approach was selected because it efficiently captures regional plume migration and pressure dissipation while remaining consistent with basin-scale project screening. Although VE models smooth fine-scale heterogeneity near wells, they provide reliable estimates of long-term trapping, as benchmarked in recent inter-comparison studies [36,37]. A 1000-year simulation horizon was adopted in line with IPCC guidelines [1] for permanence assessment and international storage regulations, which typically define storage security over centennial to millennial timescales.

### Model credibility

The validity of the modeling approach used in this study is supported by previous benchmarking and field calibration of the VE framework for conditions comparable to the Johansen formation. Studies have demonstrated that 2D VE solvers can accurately replicate key CO_2_ storage metrics, including plume extent, maximum thickness, residual saturation, and capillary transition-zone behavior, in about ±10% compared to full 3D simulations over timeframes ranging from 50 to 400 years. For example, Ligaarden and Nilsen [36] compared VE and 3D models on a sector mesh derived from the Johansen formation and observed close agreement in residual trapping and plume geometry, even with coarse vertical discretization (~20 m layers). Similarly, the Class et al. [38] benchmark showed that consistently parameterized VE models could match 3D simulator outputs for large aquifer systems. More recently, Møyner and Nilsen [37] developed a hybrid multiresolution VE-3D solver within the MRST CO_2_lab framework, achieving one to two orders of magnitude faster execution while maintaining high fidelity in pressure-saturation response for synthetic Utsira and Johansen analogues. These collective findings provide a strong foundation for the use of VE models in long-term simulations of structural, residual, and solubility trapping in buoyancy-driven aquifer systems like Johansen.

## Results

This section presents the outcomes of the Johansen VE simulations for the four injection schedules over a 1000-year horizon. Results are organized into plume migration dynamics, pressure evolution, and long-term trapping distributions.

### Plume migration dynamics

**Fig 3:**
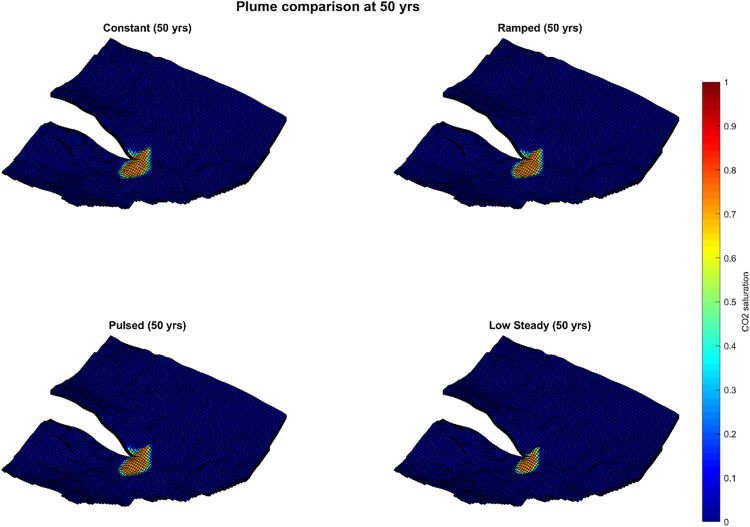
CO_2_ plume saturation at 50 years for four injection schedules.

**Fig 4:**
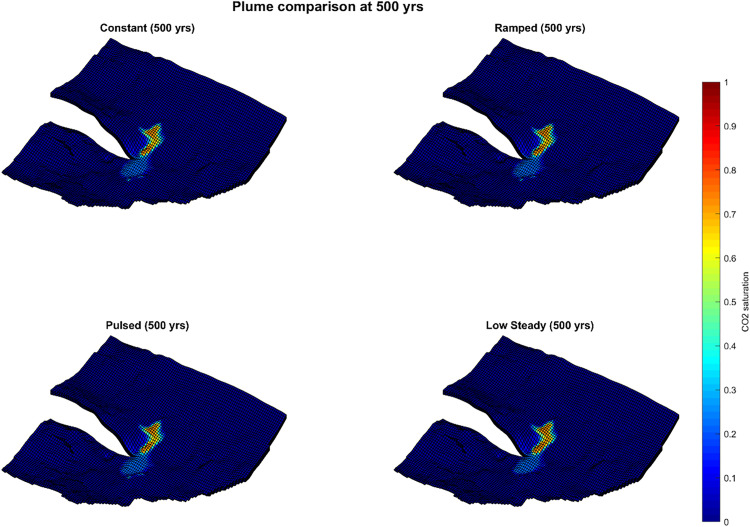
CO_2_ plume saturation at 500 years for four injection schedules.

**Fig 5:**
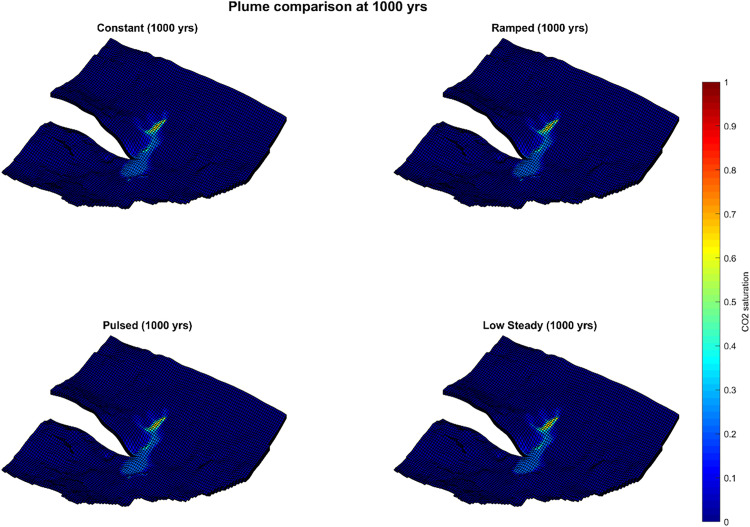
CO_2_ plume saturation at 1000 years for four injection schedules.

Plume evolution snapshots are shown in Figs 3–5, with quantitative metrics summarized in Tables 2–4. At 50 years (Fig 3, Table 2), the plume footprint varied widely among the schedules due to differences in injection rates. The constant and pulsed schedules produced footprints of ~53 km^2^, the ramped case 52 km^2^, while the low-steady schedule exhibited a much smaller footprint of 29 km^2^ since only half of its mass had been injected by that stage.

**Table 2:** CO_2_ plume footprint area at 50 years for four injection schedules.

At 50 years	Plume Area (km^2^)
**Constant**	53.27
**Ramped**	51.89
**Pulsed**	53.52
**Low-Steady**	28.68

**Table 3:** CO_2_ plume footprint area at 500 years for four injection schedules.

At 500 years	Plume Area (km^2^)
**Constant**	81.57
**Ramped**	80.73
**Pulsed**	81.82
**Low-Steady**	77.05

**Table 4:** CO_2_ plume footprint area at 1000 years for four injection schedules.

At 1000 years	Plume Area (km^2^)
**Constant**	92.27
**Ramped**	92.79
**Pulsed**	92
**Low-Steady**	89.16

By 500 years (Fig 4, Table 3), plume areas converged substantially across cases: constant 81.6 km^2^, ramped 80.7 km^2^, pulsed 81.8 km^2^, and low-steady 77.1 km^2^. At 1000 years (Fig 5, Table 4), final plume footprints were nearly identical: constant 92.3 km^2^, ramped 92.8 km^2^, pulsed 92.0 km^2^, and low-steady 89.2 km^2^. The maximum spread among schedules was < 4% of the largest footprint.

These results indicate that while scheduling strongly affects early plume development, particularly in the low-steady case, long-term plume geometry is only weakly sensitive to injection schedule. By 1000 years, plume footprints across all scenarios had essentially converged, a consequence of lateral connectivity and redistribution processes. From a monitoring perspective, this convergence implies that post-injection surveillance footprints are largely independent of operational scheduling, reducing uncertainty in the design of long-term monitoring strategies.

### Pressure evolution

**Fig 6:**
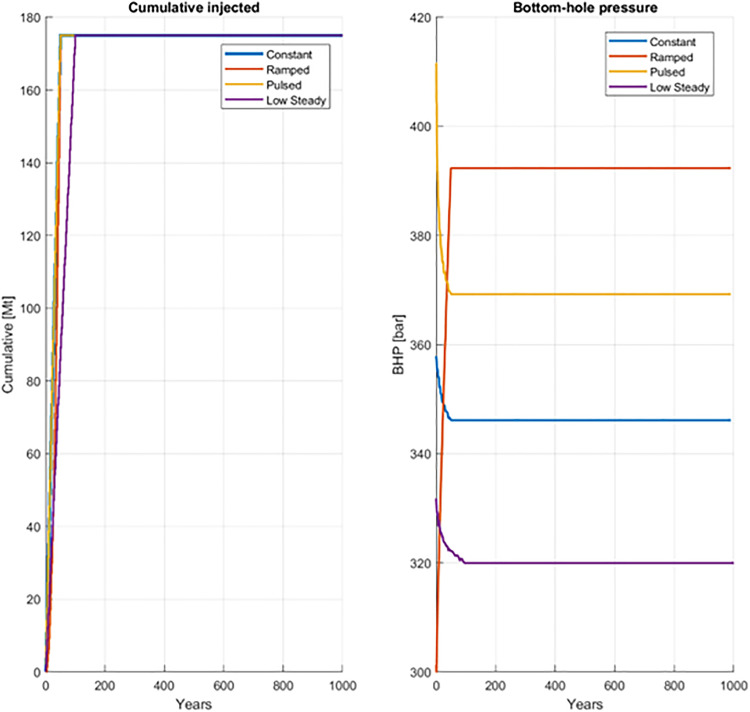
BHP histories over 1000 years for four injection schedules.

BHP histories are shown in Fig 6. Schedule strongly influenced peak injection pressures. The pulsed case produced the highest peak (≈412 bar), followed by the ramped (≈392 bar). The low-steady (≈332 bar) and constant case (≈358 bar) cases exhibited substantially lower maxima.

After injection ceased, BHP declined in most cases toward hydrostatic conditions except the ramped case, and by several centuries post-injection, pressure curves had converged. These results confirm that scheduling is an effective operational lever for managing short-term injectivity and pressure-related risks, even though long-term reservoir pressure is governed mainly by aquifer openness. Importantly, the low-steady and constant scenarios reduced peak injection pressures by up to 80 bar compared to the pulsed case, a difference large enough to influence well integrity and caprock safety margins. This highlights schedule design as a practical tool for ensuring compliance with regulatory pressure thresholds during the injection phase.

### Trapping distribution

The final mass balance after 1000 years is reported in Table 5. All cases conserved mass within tolerance (≈175 Mt injected). Dissolution dominated across all scenarios, accounting for 100.5–101.7 Mt (≈58%). Total residual trapping represented ≈30–34%, including 51–54 Mt of residual and 5–6 Mt immobilized as residual within the plume. The free plume fraction persisted at 14–16 Mt (≈8–9%).

**Table 5:** Final CO_2_ trapping inventory at 1000 years, partitioned by mechanism (free plume, residual in plume, residual, dissolved).

Injection Scenarios	Free Plume (Mt)	Residual In Plume (Mt)	Residual (Mt)	Dissolved (Mt)	Total (Mt)
**Constant**	14.5505	5.4907	53.5119	101.0040	174.5571
**Ramped**	14.5914	5.5062	53.9190	100.5288	174.5454
**Pulsed**	14.5847	5.5037	53.6020	100.8723	174.5627
**Low-Steady**	15.8625	5.9859	51.1702	101.6689	174.6875

**Fig 7:**
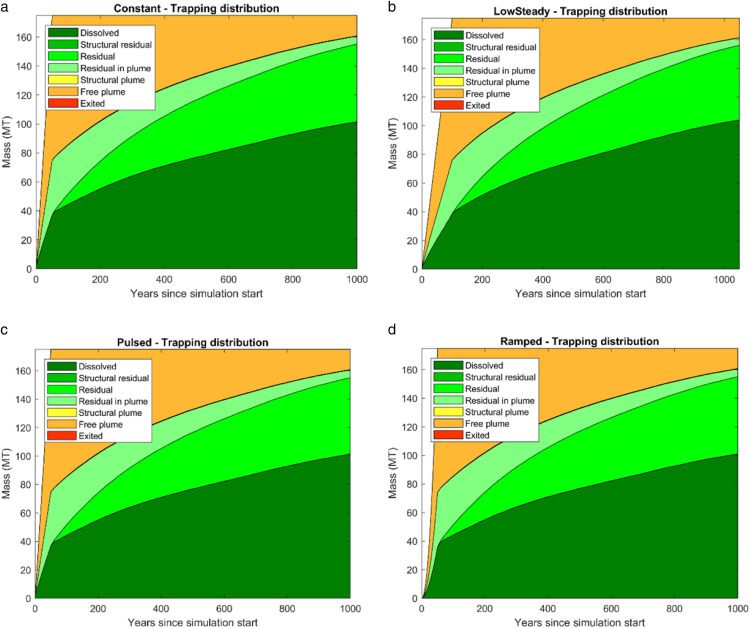
Time evolution of CO_2_ trapping mechanisms for four injection schedules.

**Fig 8:**
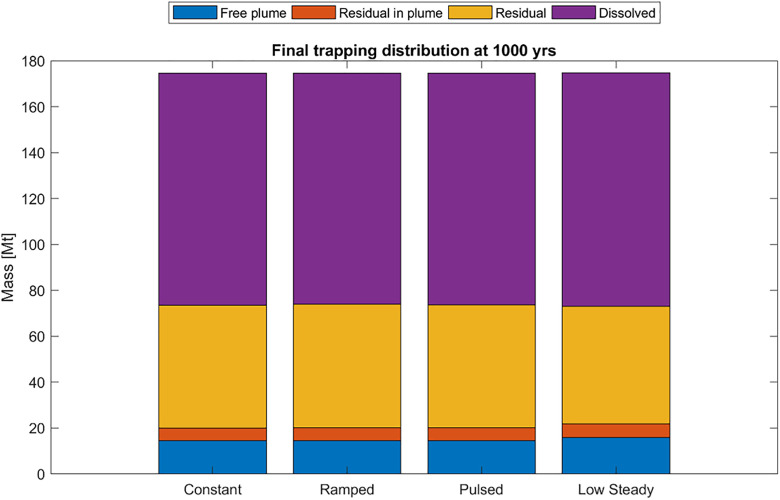
Final trapping inventory at 1000 years, shown as stacked bars.

**Fig 9:**
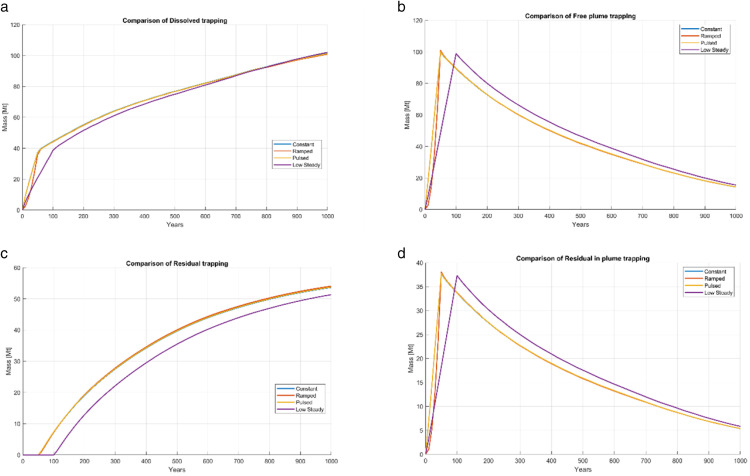
Comparison of trapping mechanisms across the four injection schedules.

Time series of trapping categories are shown in Fig 7, illustrating progressive transfer from mobile CO_2_ into dissolved and residual forms. By 1000 years, > 90% of the CO_2_ was immobilized or dissolved. Figs 8 and 9 summarize the final trapping distributions, showing near-identical patterns across injection schedules: dissolution ≈58%, total residual trapping ≈30–34%, and free plume ≈8–9%.

Differences among schedules were minor (≤2 Mt in any category, < 1.5% of total injected mass). Thus, in this Johansen VE model, long-term storage security is effectively insensitive to injection schedule, though schedules affect short-term pressure evolution.

## Discussion

This analysis extends prior work by demonstrating at field scale and over millennial horizons that injection schedule does not materially alter the final trapping distribution in the Johansen formation. Previous studies at pore- or laboratory-scale hinted at weak schedule sensitivity, but those were restricted in duration or geometry. By contrast, the present Johansen simulations show that once equal total injection mass is assumed, long-term storage outcomes converge regardless of delivery profile. Dissolution into brine emerges as the dominant sink, accounting for 57–58% of the injected CO_2_, with residual trapping contributing 30–34% and a mobile fraction of 8–9% persisting after 1000 years. These results confirm that the long-term partitioning of CO_2_ among trapping mechanisms is robust to differences in schedule design.

The weak sensitivity of trapping to injection strategy can be explained by three physical processes. Dissolution progressively transfers mobile CO_2_ into solution, ensuring that initial differences in plume geometry and saturation diminish over time. The lateral openness of the Johansen formation, together with hydrostatic boundary conditions, promotes pressure dissipation and redistribution that erases much of the early-time imprint of injection strategy. Residual trapping, governed by hysteresis and subject to a saturation ceiling, reaches a natural limit once accessible pore space has undergone drainage-imbibition cycles. Together, these mechanisms explain why observed inter-schedule differences in trapping efficiency remain below 2 Mt, less than 2% of the total injected mass.

Although injection schedules do not materially alter the millennial-scale trapping inventory, they strongly influence short-term operational behavior. The constant injection profile reduces instantaneous BHP by about 80 bar compared to the pulsed and ramped cases, providing a potential safeguard against near-wellbore pressure risks and caprock integrity concerns. The low-steady schedule, while initially associated with reduced injectivity, ultimately converges with the other cases in terms of long-term trapping and plume geometry. These outcomes suggest that schedule choice should primarily be viewed as a tool for operational flexibility and pressure management, rather than as a lever to improve long-term trapping.

The relatively small differences observed between schedules must also be interpreted within the context of model uncertainty. Benchmark comparisons between VE and full 3D models typically report deviations in about 10% or more, far exceeding the < 2% differences reported here [38,39]. This indicates that geological heterogeneity, permeability distribution, solubility kinetics, and interfacial properties exert stronger controls on storage outcomes than schedule design. Recent laboratory investigations [40] demonstrate that reductions in CO_2_-brine interfacial tension (IFT) and wettability alteration can enhance residual and solubility trapping efficiency, reinforcing the significance of interfacial effects observed here. Recent work by Mouallem et al. [41] shows that CO_2_-brine IFT varies with pressure, temperature, and salinity, influencing capillary entry pressure and plume stability. These effects are implicitly represented through the calibrated capillary-pressure functions used in this study, but explicit treatment of variable IFT could further refine predictions of trapping efficiency and migration behavior in future models. Consequently, future monitoring and modelling efforts should prioritize constraining reservoir properties, interfacial characteristics, and dissolution processes, while treating scheduling as a secondary factor for millennial-scale security.

The predicted partitioning of CO_2_ among dissolved, residual, and mobile states also has implications for monitoring and verification. Dissolution, which dominates the mass balance, enhances storage security but is difficult to verify directly, as dissolved CO_2_ produces weak geophysical signals. Residual trapping, while accounting for roughly a third of the mass, is similarly difficult to observe since it occurs at the pore scale. By contrast, the free plume-although reduced to less than 10% after 1000 years-remains detectable with seismic imaging and thus represents the most practical monitoring target. This suggests that monitoring priorities will evolve over time: during injection and shortly thereafter, emphasis should be on plume imaging and migration pathways, while longer-term assessment will require indirect approaches such as pressure surveillance, geochemical sampling, or tracer studies. The persistence of a small mobile fraction underscores the likelihood that monitoring obligations will extend far beyond the active injection phase, especially under regulatory frameworks requiring proof of long-term containment.

Taken together, these findings highlight a tension between the processes that secure CO_2_ in the subsurface and the evidence available to verify them. While dissolution and residual trapping provide permanence, they are not easily accessible to direct observation, reinforcing the importance of integrated monitoring strategies that combine geophysical, geochemical, and modelling approaches.

Finally, the transferability of these findings should be considered. In large, laterally extensive saline aquifers with open boundaries and high solubility potential, schedule effects are expected to remain secondary. However, in compartmentalized formations with restricted transmissivity or in systems where solubility is limited, plume persistence could magnify schedule-related differences. Hybrid VE-3D approaches near injection wells will also be valuable for resolving local trapping and pressure responses that are smoothed in vertically integrated models. Overall, the Johansen results indicate that in well-connected, laterally open saline aquifers, schedule effects on long-term trapping remain secondary to geological and thermodynamic controls. However, in more compartmentalized or heterogeneous formations, the influence of scheduling could become more significant, highlighting the need for site-specific assessments.

## Conclusions

This study provides the long-horizon, equal-mass comparison of operationally relevant injection schedules in a field-calibrated Johansen model. Using vertically integrated simulations extended over 1000 years, the analysis shows that long-term CO_2_ partitioning is remarkably robust to injection schedule. Across all cases, approximately 57–58% of the injected mass dissolves into brine, 30–34% is immobilized by residual trapping, and 8–9% remains as a mobile plume, with inter-schedule differences of less than 2 Mt-well within expected model uncertainty. This finding establishes that, at least for an open and laterally connected aquifer such as Johansen, the ultimate trapping balance is governed primarily by reservoir properties and dissolution dynamics, not by the choice of injection schedule.

From an operational perspective, however, scheduling exerts a strong influence on near-term injectivity and wellbore pressure. Peak bottom-hole pressures differed by about 80 bar between the pulsed, ramped, and constant-rate scenarios, a difference large enough to affect well integrity and caprock safety margins. This demonstrates that injection scheduling should be treated as a practical lever for managing pressure risk and maintaining injectivity, rather than as a strategy for altering long-term storage performance.

For CO_2_ storage strategy more broadly, these results highlight the distinction between operational and geological controls on security. Long-term permanence is dictated by dissolution and residual trapping, which are robust to schedule choice but difficult to verify directly with conventional monitoring. Operators and regulators should therefore prioritize reservoir characterization, solubility kinetics, and integrated monitoring approaches over attempts to optimize injection profiles. Schedule design remains valuable for ensuring safe and efficient injection operations, but not for guaranteeing millennial-scale containment.

In practical terms, these findings offer guidance for the design and operation of large-scale CO_2_ storage projects. By clarifying that long-term trapping in well-connected saline aquifers is relatively robust to scheduling, injection profiles can be selected to balance short-term pressure management with infrastructure constraints. This understanding supports more flexible and cost-effective project planning while maintaining long-term containment integrity. Future research should focus on hybrid VE-3D modeling near injection wells, accounting for variable interfacial properties, and validating schedule effects with field-scale monitoring data to enhance predictive reliability and risk assessment.

## Abbreviations:

BHP: bottom hole pressure
CCS: carbon capture and storage
CO_2_: carbon dioxide
IFT: interfacial tension
MRST: MATLAB reservoir simulation toolbox
VE: vertical equilibrium
2D: two-dimensional
3D: three-dimensional
